# Reduction in GLP-1 secretory capacity may be a novel independent risk factor of coronary artery stenosis

**DOI:** 10.1038/s41598-021-95065-9

**Published:** 2021-08-02

**Authors:** Chihiro Nagase, Masaya Tanno, Hidemichi Kouzu, Takayuki Miki, Junichi Nishida, Naoto Murakami, Nobuaki Kokubu, Nobutaka Nagano, Ryo Nishikawa, Nobuhiro Yoshioka, Tohru Hasegawa, Hiroyuki Kita, Akihito Tsuchida, Hirofumi Ohnishi, Tetsuji Miura

**Affiliations:** 1 Department of Cardiovascular, Renal and Metabolic Medicine, Sapporo Medical University, Sapporo, Japan; 2 Sapporo Circulation Hospital, Sapporo, Japan; 3 Department of Cardiology, JR Sapporo Hospital, Sapporo, Japan; 4 Department of Cardiology, JCHO Hokushin Hospital, Sapporo, Japan; 5 Department of Public Health, Sapporo Medical University, Sapporo, Japan; 6 Department of Clinical Pharmacology, Faculty of Pharmaceutical Sciences, Hokkaido University of Science, 15-4-1, Maeda-7, Teine-ku, Sapporo, Japan

**Keywords:** Atherosclerosis, Coronary artery disease and stable angina

## Abstract

Multiple factors regulate glucagon-like peptide-1 (GLP-1) secretion, but a group of apparently healthy subjects showed blunted responses of GLP-1 secretion in our previous study. In this study, we examined whether the reduction in GLP-1 secretory capacity is associated with increased extent of coronary artery stenosis in non-diabetic patients. Non-diabetic patients who were admitted for coronary angiography without a history of coronary interventions were enrolled. Coronary artery stenosis was quantified by Gensini score (GS), and GS ≥ 10 was used as an outcome variable based on its predictive value for cardiovascular events. The patients (mean age, 66.5 ± 8.8 years; 71% males, n = 173) underwent oral 75 g-glucose tolerant tests for determination of glucose, insulin and active GLP-1 levels. The area under the curve of plasma active GLP-1 (AUC-GLP-1) was determined as an index of GLP-1 secretory capacity. AUC-GLP-1 was not correlated with fasting glucose, AUC-glucose, serum lipids or indices of insulin sensitivity. In multivariate logistic regression analysis for GS ≥ 10, AUC-GLP-1 < median, age and hypertension were selected as explanatory variables, though fasting GLP-1 level was not selected. The findings suggest that reduction in GLP-1 secretory capacity is a novel independent risk factor of coronary stenosis.

## Introduction

Although the incidence of acute coronary syndrome and its mortality have been declining in the past few decades, acute myocardial infarction is still a leading cause of death worldwide. Control of major risk factors of atherosclerotic diseases, i.e., smoking, hypertension, dyslipidemia and diabetes mellitus, is a fundamental strategy for primary and secondary prevention of coronary artery diseases. Recent development of pharmacological agents such as PCSK9 antibody and SGLT2 inhibitors have provided multiple options for control of risk factors in patients. On the other hand, more than 20% of patients with ST-elevation myocardial infarction (STEMI) do not have major risk factors of atherosclerosis^1,2^, and a significant proportion of patients with prior myocardial infarction have recurrent cardiovascular events despite guideline-based control of the risk factors, indicating the presence of “residual” risks^3,4^.

As “residual” risk factors of atherosclerosis, inflammation, residual lipid abnormalities, and residual thrombosis risk have been characterized by observational studies and clinical trials^5–8^. Recently, pleiotropic effects of glucagon-like peptide-1 (GLP-1), an incretin, have received attention not only for the benefit of GLP-1 analogues in management of diabetes^9,10^ but also for possible involvement in etiologies of various diseases including hepatic steatosis, renal failure and inflammatory bowel diseases^11–13^. GLP-1 receptors are expressed in vascular endothelial cells and smooth muscle cells^9^, and experiments using animal models have shown that treatment with a GLP-1 receptor agonist affords protective effects on vascular endothelial cells and attenuates intimal hyperplasia and vascular stenosis after vascular injury^14–16^. On the other hand, our previous cohort study showed that there is a group of apparently healthy subjects with a blunted response of GLP-1 secretion to oral glucose loading and that their blood pressure was inversely correlated with the response of GLP-1^17^. Interestingly, plasma GLP-1 level at baseline or its change after oral glucose loading was not correlated with insulin sensitivity, plasma glucose, or serum lipids^17^. Based on the findings^14–17^, we hypothesized that the low response of GLP-1 to feeding is a residual risk factor of coronary atherosclerosis. To test this hypothesis, we prospectively examined the relationship between GLP-1 secretion capacity and extent of coronary artery stenosis in non-diabetic patients in the present study.

## Methods

This study was approved by the internal review boards in Sapporo Medical University Hospital and its three affiliated hospitals, Sapporo Circulation Hospital, JR Sapporo Hospital, JCHO Hokushin Hospital, and registered as Clinical Trials.gov Identifier: NCT02280837. This study was conducted in accordance with the World Medical Association Declaration of Helsinki and as a project of the BOREAS (Broad-range Organization for REnal, Arterial and cardiac studies by Sapporo Medical University Affiliates) Study.

### Study subjects

Patients who met all inclusion criteria on admission were consecutively enrolled from March 2015 to March 2019. The inclusion criteria were non-diabetic patients at ages of 35–80 years, no change in therapy for dyslipidemia within 3 months before admission and scheduled coronary angiography (CAG) to assess coronary artery stenoses. Exclusion criteria were HbA1c level ≥ 6.5%, diabetes mellitus with insulin or oral anti-diabetic agent treatment, indication for immediate additional medical treatment for coronary artery disease, indication for emergency coronary intervention, and prior history of percutaneous coronary intervention. Patients who met at least one of the exclusion criteria were not enrolled in this study. Written informed consent was obtained from all enrolled study subjects.

### Protocol of the study

Eligibility of patients for enrollment was examined on day 1 of hospital admission. The enrolled study subjects underwent standard blood tests on day 1 or 2 after admission, an oral glucose tolerance test on day 2 or 3, and CAG on day 3 or a day no later than day 14. Since treatment or checking for a non-cardiac disease before CAG can delay the schedule of CAG, we set the timing of CAG at 3–14 days after admission, though CAG was performed in most cases within 5 days after admission.

### Oral glucose tolerance test (OGTT)

After fasting for more than 10 h, the subjects underwent an oral 75 g-OGTT. Venous blood was sampled before and 30 min, 60 min, and 120 min after oral 75 g glucose loading for determination of levels of glucose, immunoreactive insulin (IRI), and active GLP-1 (GLP-1[7–36] amide and GLP-1[7–37]). Glucose and IRI were determined by the hexokinase method and enzyme immunoassay, respectively. Blood samples for GLP-1 assay were collected in tubes containing a DPP-4 inhibitor (BD P700, Becton, Dickinson and Co.). Plasma active GLP-1 level was determined by using an ELISA kit (GLP-1 Active ELISA kit, EGLP-35k, Millipore Inc.) that measures active GLP-1 without cross-reacting with the inactive form of GLP-1[9–36]. Capacity of GLP-1 secretion was determined as the area under the curve (AUC) of plasma active GLP-1 level (AUC-GLP-1) in the OGTT by use of the trapezoid rule. Similarly, AUC of plasma glucose (AUC-PG) and AUC of IRI (AUC-IRI) were also calculated. Homeostasis model assessment-β (HOMA-β) was calculated as an index of insulin secretion^18^.

### Assessment of insulin sensitivity and renal function

HOMA of insulin resistance (HOMA-IR) and Matsuda-DeFronzo index were calculated as indices of insulin sensitivity^18,19^. Renal function was assessed by serum creatinine and estimated glomerular filtration rate (eGFR) calculated by an equation for the Japanese population^20^.

### Assessment of severity of coronary stenosis

Coronary artery stenosis was quantified by Gensini score^21^ as in our previous study^22^ and studies in which the impact of non-obstructive coronary lesions on prognosis was examined^23,24^. Gensini score grades narrowing of the lumen as follows: 1, 1–25% stenosis; 2, 26–50% stenosis; 4, 51–75% stenosis; 8, 76–90% stenosis; 16, 91–99% stenosis; and 32, total occlusion. This score is multiplied by a factor accounting for the importance of the lesion position in the coronary arterial tree: 5 for the left main trunk, 2.5 for the proximal left anterior descending artery, 2.5 for the proximal left circumflex artery (3.5 in the case of left dominance) and 1 for the proximal right coronary artery. The severity of the disease was expressed as the sum of the scores for individual lesions. Gensini score was calculated by observers who were blinded to the results of biochemical examinations. Results of CAG performed in the affiliated hospitals were sent to a group in Sapporo Medical University Hospital for grading Gensini score.

In the present study, we used Gensini score ≥ 10 as an outcome variable. The rationale for selection of the outcome variable is twofold. First, Gensini score data were not normally distributed (see “Results”), thus being unsuitable for parametric analysis. Second, results of earlier studies^23,24^ suggested that Gensini score ≥ 10 is associated with a significantly increased risk of cardiovascular events, though Gensini score ≥ 10 does not necessarily mean presence of > 50% coronary stenosis.

### Statistical analysis

Numeric variables are expressed as means ± SD or medians (interquartile ranges). The distribution of each parameter was tested for its normality by using the Shapiro–Wilk W test. Differences in parameters between groups were tested by one-way analysis of variance, the Kruskal–Wallis test or χ^2^ test where appropriate. Relationships between parameters were examined by calculation of Spearman’s rank correlation coefficients and by multivariate logistic regression analysis. In multivariate logistic analysis, models were prepared by forward selection of explanatory variables, and better fit models were selected by using Akaike Information Criterion (AIC). A p value of less than 0.05 was considered statistically significant. All data were analyzed by using JMP pro 15.1.0 (SAS Institute, Cary, NC).

## Results

### Clinical characteristics of the subjects

During the enrollment period, 190 patients were eligible for this study and they completed the protocol. Unfortunately, because of unpredicted trouble in the storage of blood samples (mechanical failure to keep the temperature of the sample at less than 4 degrees Celsius) in one institute, samples from 17 patients became unsuitable for analyses. Thus, data for 173 subjects contributed to the present analyses. Baseline clinical characteristics of the subjects are summarized in Table 1. There were significant differences between males and females in age (65.6 ± 8.7 vs. 68.9 ± 8.5 years), body mass index (BMI) (24.5 ± 3.6 vs. 22.9 ± 3.3), AUC of plasma glucose (1896 ± 365 vs. 1731 ± 414), serum levels of LDL-cholesterol (106 ± 24 vs. 118 ± 35 mg/dl), HDL-cholesterol (48 ± 12 vs. 56 ± 13 mg/dl), triglycerides (139 ± 63 vs. 108 ± 53 mg/dl) and creatinine (0.91 ± 0.20 vs. 0.71 ± 0.18 mg/dl).

**Table 1:** Baseline characteristics of patients.

	Overall (n = 173)	Male (n = 122)	Female (n = 51)	p
Age (years)	66.5 ± 8.8	65.6 ± 8.7	68.9 ± 8.5	0.026
BMI (kg/m^2^)	24.1 ± 3.6	24.5 ± 3.2	22.9 ± 3.3	0.006
sBP (mmHg)	123.4 ± 17.3	124.5 ± 16.6	121.0 ± 18.6	0.222
Hypertension (%)	114 (66)	85 (70)	29 (57)	0.105
Fasting PG (mg/dl)	90.4 ± 10.4	91.2 ± 11.0	88.4 ± 8.4	0.115
AUC-PG (a.u.)	1847 ± 387	1896 ± 365	1731 ± 414	0.010
Fasting IRI (μIU/ml)	5.4 ± 3.6	5.4 ± 3.7	5.4 ± 3.4	0.906
AUC-IRI (a.u.)	585 ± 456	593 ± 466	565 ± 432	0.713
Fasting GLP-1 (pmol/l)	1.0 (1.0–2.3)	1.0 (1.0–2.0)	1.0 (1.0–2.3)	0.389
AUC-GLP-1 (a.u.)	447 (271–806)	411 (244–756)	544 (345–1049)	0.039
LDL-C (mg/dl)	110 ± 28	106 ± 24	118 ± 35	0.017
HDL-C (mg/dl)	50 ± 13	48 ± 12	56 ± 13	< 0.001
Triglyceride (mg/dl)	130 ± 62	139 ± 63	108 ± 53	0.003
S-Cre (mg/dl)	0.85 ± 0.22	0.91 ± 0.20	0.71 ± 0.18	< 0.001
eGFR (mL/min/1.73 m^2^)	67.7 ± 17.1	67.9 ± 16.0	67.4 ± 19.5	0.870
HbA1c (%)	5.7 ± 0.4	5.7 ± 0.4	5.7 ± 0.3	0.941
U-Alb/U-Cre (mg/gCre)	11.9 (4.4–70.9)	12.1 (4.0–90.5)	11.8 (4.6–55.3)	0.345
Matsuda-DeFronzo index	10.0 ± 9.3	10.2 ± 10.0	9.6 ± 7.5	0.704
HOMA-IR	1.2 ± 0.8	1.2 ± 0.9	1.2 ± 0.8	0.707
HOMA-β	77.3 ± 51.1	76.5 ± 52.6	79.1 ± 47.7	0.765
Gensini score	10.8 (4.1–25.8)	13.5 (5.0–26.0)	7.0 (1.5–18.0)	0.060

sBP: systolic blood pressure; BMI: body mass index; PG: plasma glucose; IRI: immunoreactive insulin; LDL-C: LDL cholesterol; HDL-C: HDL cholesterol; S-Cre: serum creatinine; eGFR: estimated glomerular filtration rate; HbA1c: hemoglobin A1c; U-Alb/U-Cre: ratio of urinary albumin to urinary creatinine.

Data are means ± SD or medians (interquartile ranges).

While fasting GLP-1 levels were not different between males and females, the AUC-GLP-1 value was lower in males than in females: 411 [244–756] vs. 544 [345–1049] (Table 1). Gensini score in overall subjects was 10.8 [4.1–25.8], and it tended to be larger in males than in females (13.5 [5.0–26.0] vs. 7.0 [1.5–18.0]), though the difference was not statistically significant.

### Relationships between GLP-1 secretory function and metabolic parameters

Because data for fasting GLP-1 level and AUC-GLP-1 were not normally distributed, their correlations with metabolic parameters were examined by Spearman’s rank correlation coefficients. Fasting GLP-1 and AUC-GLP1 were not significantly correlated with age, BMI, levels of plasma glucose, IRI, and serum lipids, indices of insulin sensitivity, or indices of renal function (Table 2). As expected, there was a significant correlation between fasting GLP-1 level and AUC-GLP-1.

**Table 2:** Correlation coefficients for fasting GLP-1 and AUC-GLP-1.

	Fasting GLP-1		AUC-GLP-1	
	Spearman's ρ	p	Spearman's ρ	p
Age (years)	− 0.029	0.705	− 0.017	0.828
BMI (kg/m^2^)	0.037	0.632	− 0.047	0.539
sBP (mmHg)	− 0.100	0.191	− 0.072	0.348
Fasting PG (mg/dl)	0.124	0.105	0.072	0.346
AUC-PG (a.u.)	0.045	0.561	− 0.122	0.112
Fasting IRI (μIU/ml)	0.129	0.096	0.143	0.064
AUC-IRI (a.u.)	0.072	0.559	0.067	0.390
Fasting GLP-1 (pmol/l)			0.348	< 0.001
AUC-GLP-1 (a.u.)	0.348	< 0.001		
LDL-C (mg/dl)	− 0.102	0.181	− 0.024	0.752
HDL-C (mg/dl)	− 0.141	0.064	0.081	0.291
Triglyceride (mg/dl)	− 0.030	0.698	− 0.019	0.809
S-Cre (mg/dl)	0.026	0.734	− 0.102	0.183
eGFR (mL/min/1.73 m^2^)	− 0.003	0.970	0.023	0.762
HbA1c (%)	− 0.023	0.765	− 0.043	0.574
log U-Alb/U-Cre (mg/gCre)	− 0.103	0.181	− 0.127	0.100
Matsuda-DeFronzo index	− 0.150	0.052	− 0.121	0.117
HOMA-IR	0.142	0.066	0.146	0.059
HOMA-β	0.059	0.452	0.071	0.359
Gensini score	− 0.085	0.298	− 0.105	0.198

sBP: systolic blood pressure; BMI: body mass index; PG: plasma glucose; IRI: immunoreactive insulin; LDL-C: LDL cholesterol; HDL-C: HDL cholesterol; S-Cre: serum creatinine; eGFR: estimated glomerular filtration rate; HbA1c: hemoglobin A1c; U-Alb/U-Cre: ratio of urinary albumin to urinary creatinine.

### Comparison of clinical characteristics between high AUC-GLP-1 and low AUC-GLP-1

Table 3 shows a comparison of clinical characteristics in subjects with AUC-GLP-1 above the median (high AUC-GLP-1 group) and those with AUC-GLP-1 below the median (low AUC-GLP-1 group). The low AUC-GLP-1 group had higher systolic blood pressure than the high AUC-GLP-1 group, which finding was consistent with results of our previous study^17^. Fasting GLP-1 level and HOMA-IR were higher in the high AUC-GLP-1 group than in the low AUC-GLP-1 group, while there was no significant difference in AUC-PG, AUC-IRI, serum lipid levels or Matsuda-DeFronzo index between the two groups. Interestingly, Gensini score was significantly smaller in the high AUC-GLP-1 group than in the low AUC-GLP-1 group.

**Table 3:** Baseline characteristics of patients grouped according to AUC-GLP-1 being higher or lower than its median value.

	High AUC-GLP-1(n = 86)^a^	Low AUC-GLP-1(n = 87)^a^	p
Age (years)	66.1 ± 8.8	67.0 ± 8.8	0.522
Male (%)	56 (65.1)	66 (75.9)	0.093
BMI (kg/m^2^)	24.0 ± 3.8	24.2 ± 3.3	0.652
sBP (mmHg)	120.6 ± 16.9	125.8 ± 17.2	0.049
Hypertension (%)	55 (64)	59 (68)	0.519
Fasting PG (mg/dl)	90.9 ± 9.1	89.8 ± 11.6	0.507
AUC-PG (a.u.)	1811 ± 383	1881 ± 390	0.236
Fasting IRI (μIU/ml)	5.9 ± 3.8	4.8 ± 3.2	0.050
AUC-IRI (a.u.)	633 ± 506	532 ± 396	0.153
Fasting GLP-1 (pmol/l)	1.0 (1.0–2.5)	1.0 (1.0–1.0)	< 0.001
AUC-GLP-1 (a.u.)	803 (633–1322)	273 (173–373)	< 0.001
LDL-C (mg/dl)	106 ± 27	113 ± 30	0.119
HDL-C (mg/dl)	52 ± 13	49 ± 12	0.178
Triglyceride (mg/dl)	128 ± 61	130 ± 61	0.887
S-Cre (mg/dl)	0.84 ± 0.25	0.86 ± 0.19	0.534
eGFR (mL/min/1.73 m^2^)	68.5 ± 18.1	67.0 ± 16.2	0.586
HbA1c (%)	5.7 ± 0.4	5.7 ± 0.4	0.845
U-Alb/U-Cre (mg/gCre)	10.0 (4.6–50.0)	15.2 (4.0–154.3)	0.619
Matsuda-DeFronzo index	9.4 ± 9.7	10.8 ± 9.0	0.319
HOMA-IR	1.3 ± 0.9	1.1 ± 0.8	0.044
HOMA-β	79.8 ± 50.5	74.1 ± 51.9	0.469
Gensini score	7.5 (2.4–23.1)	15.5 (5.5–26.0)	0.040

sBP: systolic blood pressure; BMI: body mass index; PG: plasma glucose; IRI: immunoreactive insulin; LDL-C: LDL cholesterol; HDL-C: HDL cholesterol; S-Cre: serum creatinine; eGFR: estimated glomerular filtration rate; HbA1c: hemoglobin A1c; U-Alb/U-Cre: ratio of urinary albumin to urinary creatinine.

Data are means ± SD or medians (interquartile ranges).

^a^Patients with higher or lower level of AUC-GLP-1 than median value (446.5).

### Relationships between high Gensini score and metabolic parameters

Table 4 shows comparisons of clinical characteristics between subjects with Gensini score < 10 and those with Gensini score ≥ 10. Age and systolic blood pressure were higher and hypertension and subjects with AUC-GLP-1 below median were more frequent in the group with Gensini score ≥ 10 than those in the group with Gensini score < 10. Levels of fasting plasma glucose, IRI, serum lipids or indices of insulin resistance were not significantly different between the two groups (Table 4).

**Table 4:** Baseline characteristics of patients grouped according to Gensini score being < 10 or ≥ 10.

	GS < 10 (n = 70)	GS ≥ 10 (n = 82)	p
Age (years)	65.1 ± 9.7	67.9 ± 7.5	0.042
Male (%)	47 (64)	60 (77)	0.070
BMI (kg/m^2^)	23.7 ± 3.9	24.5 ± 3.4	0.159
sBP (mmHg)	119 ± 17	127 ± 18	0.012
Hypertension (%)	42 (57)	57 (73)	0.035
Fasting PG (mg/dl)	89.5 ± 9.1	90.4 ± 11.0	0.579
AUC-PG (a.u.)	1812 ± 348	1872 ± 420	0.339
Fasting IRI (μIU/ml)	5.7 ± 3.8	5.8 ± 3.5	0.787
AUC-IRI (a.u.)	637 ± 470	616 ± 459	0.779
Fasting GLP-1 (pmol/l)	1.0 (1.0–2.3)	1.0 (1.0–2.0)	0.280
AUC-GLP-1 (a.u.)	567 (329–912)	407 (269–793)	0.165
AUC-GLP-1 < median (%)	28 (38)	45 (58)	0.011
LDL-C (mg/dl)	108 ± 29	110 ± 29	0.560
HDL-C (mg/dl)	51 ± 13	49 ± 12	0.600
Triglyceride (mg/dl)	120 ± 62	138 ± 60	0.079
S-Cre (mg/dl)	0.85 ± 0.24	0.86 ± 0.20	0.904
eGFR (mL/min/1.73 m^2^)	67.0 ± 18.2	67.7 ± 16.2	0.813
HbA1c (%)	5.7 ± 0.4	5.7 ± 0.4	0.657
U-Alb/U-Cre (mg/gCre)	8.0 (4.3–31.6)	8.6 (3.8–25.9)	0.623
Matsuda-DeFronzo index	8.6 ± 6.1	8.8 ± 7.4	0.899
HOMA-IR	1.3 ± 0.9	1.3 ± 0.8	0.747
HOMA-β	81.2 ± 52.9	83.9 ± 50.2	0.753
Gensini score	4.0 (1.0–7.0)	24.3 (16.0–34.6)	< 0.001

GS: Gensini score; sBP: systolic blood pressure; BMI: body mass index; PG: plasma glucose; IRI: immunoreactive insulin; LDL-C: LDL cholesterol; HDL-C: HDL cholesterol; S-Cre: serum creatinine; eGFR: estimated glomerular filtration rate; HbA1c: hemoglobin A1c; U-Alb/U-Cre: ratio of urinary albumin to urinary creatinine.

Data are means ± SD or medians (interquartile ranges).

Multivariate logistic analysis for Gensini score ≥ 10 was performed by using sex, age, BMI, HOMA-IR, history of hypertension, LDL-cholesterol, HDL-cholesterol, eGFR, and high AUC-GLP-1 (i.e., AUC-GLP-1 > median) as possible explanatory variables, and better fit models were selected by using AIC. As shown in Table 5, low AUC-GLP-1 was selected as an explanatory variable for Gensini score ≥ 10 with significantly high odds ratio in Model 1 in which sex, age and hypertension were included as explanatory variables. Low AUC-GLP-1 had a significantly high odds ratio for Gensini score ≥ 10 also in Model 2 in which sex, age, hypertension and eGFR were incorporated as other variables. We additionally performed analyses by dividing the study subjects according to tertiles of AUC-GLP-1 (i.e., AUC-GLP-1 < 380, 380–472, and > 472). Odds ratios for Gensini score ≥ 10 were higher in low and middle AUC-GLP-1 tertiles than in high AUC-GLP-1 tertile in both Model 1 and Model 2 (Supplementary Table 1), but the differences did not reach statistical significance presumably because of small number of study subjects in each tertile.

**Table 5:** Multivariate logistic regression analysis for GS ≥ 10.

	B	SE	Wald χ^2^	OR	95%CI		p
**Model 1**							
Sex	0.39	0.20	3.74	2.17	0.99	4.77	0.053
Age	− 0.04	0.02	3.29	1.04	1.00	1.08	0.070
AUC-GLP-1 < median	0.37	0.17	4.56	2.11	1.06	4.19	0.033
Hypertension	0.36	0.19	3.61	2.04	0.98	4.26	0.057
**Model 2**							
Sex	0.39	0.20	3.78	2.18	0.99	4.80	0.052
Age	− 0.05	0.02	4.60	1.05	1.00	1.05	0.032
AUC-GLP-1 < median	0.39	0.18	4.83	2.18	1.09	4.35	0.028
Hypertension	0.41	0.19	4.35	2.28	1.07	4.89	0.031
eGFR	− 0.02	0.01	2.13	0.98	0.96	1.01	0.141

OR: odds ratio; CI: confidence interval; LDL-C: LDL cholesterol; eGFR: estimated glomerular filtration rate.

Multivariate logistic analysis for Gensini score ≥ 10 was performed by using sex, age, BMI, HOMA-IR, history of hypertension, LDL-cholesterol, HDL-cholesterol, eGFR, and AUC-GLP-1 level < median as possible explanatory variables, and better fit models were selected by using Akaike Information Criterion.

In contrast to AUC-GLP-1, fasting GLP-1 level ≤ 1 pmol/l, a level below or at lower limit of assay sensitivity, was not selected as an explanatory variable for Gensini score ≥ 10 in multivariate logistic analysis in which sex, age, BMI, HOMA-IR, history of hypertension, LDL-cholesterol, HDL-cholesterol and eGFR were used as other possible variables (Supplementary Table 2). There was no significant difference in clinical characteristics in subjects with fasting GLP-1 level > 1 pmol/l and those with fasting GLP-1 level ≤ 1 pmol/l (Supplementary Table 3).

## Discussion

To our knowledge, there have been few studies in which the relationship between plasma level of GLP-1 and atherosclerotic change in the coronary artery was examined. Recently, Mitsuhashi et al.^25^ examined GLP-1 secretion during the OGTT in patients with acute coronary syndrome (n = 85) and found that the percentage of lipid area in the plaque in a culprit vessel was significantly higher in patients with a low tertile of AUC-GLP-1 (290 ± 84). However, patients in the low AUC-GLP-1 tertile group were younger and had higher levels of LDL-cholesterol and plasma insulin than patients in the high AUC-GLP-1 tertile group. Such differences in risk factors in the low AUC-GLP-1 group obscure the role of low GLP-1 secretory response in the formation of lipid-rich coronary artery plaques. As in our previous study^17^, we found that AUC-GLP-1 was not correlated with fasting plasma glucose, serum lipids or indices of insulin resistance in the present study (Table 2), while clinical characteristics in study subjects were different between the two studies (i.e., apparently healthy subjects in a cohort vs. patients suspected of having coronary artery disease). Furthermore, we found that low AUC-GLP-1 was independently associated with an increased odds ratio for Gensini score ≥ 10 by more than two-fold after adjusting for risk factors (Table 5). The advantage of Gensini score is that it enables quantification of a wide range of extents of narrowing and length of the coronary stenosis by a score that ranges from 0 to 688. Gensini score ≥ 10 includes non-obstructive coronary lesions, but a predictive value of a score ≥ 10 is supported by the earlier findings. In a study by Yokokawa et al.^24^, the cardiovascular event rate after percutaneous coronary intervention (PCI) for ischemic heart failure was higher in patients with residual lesions of Gensini score ≥ 10. In the AtheroGene study^23^ in which a group of patients with non-obstructive coronary artery disease and a group of patients with obstructive coronary artery disease were compared, Gensini score in the non-obstructive group was 4.1 ± 2.8 (SD), indicating that 95% of the patients had a score < 9.7. The cardiovascular event rate during a median follow-up period of 4.9 years was significantly lower in the non-obstructive group than in the obstructive group^23^. Nevertheless, the present findings support the notion that blunted response of GLP-1 secretion is an independent risk factor of the coronary artery disease.

Because of the cross-sectional analysis, the present study has not clarified whether the blunted response of GLP-1 secretion is one of etiological causes of coronary artery stenosis. However, several lines of circumstantial evidence suggest the presence of such a causal relationship. GLP-1 receptor has been suggested to localize in endothelial cells and vascular smooth muscle cells^9^, though there are discrepancies in the findings possibly due to species difference and/or presence of underlying disease^26^. Animal experiments showed that GLP-1 receptor agonists suppressed neointima proliferation and narrowing of the lumen after vascular injury via endothelial nitric oxide in vivo^14–16^. Experiments in vitro demonstrated that GLP-1 suppresses inflammatory cytokine-induced intracellular signaling in endothelial cells and vascular smooth muscle cells^16^. In addition, intestinal inflammation and microbes, which potentially contribute to the progression of atherosclerosis in arteries^27^, have been shown to be modulated by activation of the GLP-1 receptor in intraepithelial lymphocytes and enteroendocrine cells^9^. Furthermore, randomized clinical trials demonstrated that treatment with GLP-1 receptor agonists reduces atherosclerotic cardiovascular events in high risk diabetic patients^28–32^. The findings in both animal experiments and clinical studies suggest that GLP-1 protects the artery from atherosclerotic changes by direct and indirect mechanisms. The results of the present study suggest the importance of physiological level of endogenous GLP-1 in prevention of coronary atherosclerosis.

AUC-GLP-1 values in the subjects in the present study ranged widely as in earlier studies^11,25^. Since there are no established data for a normal range of AUC-GLP-1 value during the OGTT, the extent of change in GLP-1 secretory capacity is difficult to determine by use of OGTT data. However, Bernsmeier et al.^11^ reported GLP-1 levels during OGTTs in 50 healthy subjects as controls for patients with non-alcoholic fatty liver. They conducted the OGTT by using almost the same blood sampling protocol as that used in our study, and AUC-GLP-1 in the normal control group was 969 ± 34 (mean ± SD, pmol/l ⨯ min). Mitsuhashi et al.^25^ reported data for AUC-GLP-1 tertiles in non-diabetic patients with acute coronary syndrome (n = 84): AUC < 395 (290 ± 84), AUC 395–660 (493 ± 73), and AUC > 660 (1,668 ± 1,229). In their study, the group with AUC < 395 had more lipid-rich coronary plaque than that in the other groups. In the present study, AUC-GLP-1 was 677 ± 637 in the 173 subjects and the median value was 446.5 (Table 1). In light of the reported range of data for AUC-GLP-1 and the presence or absence of coronary pathology, it is tempting to speculate that AUC-GLP1 < median value (i.e., 446.5) in the present study reflects insufficiency of GLP-1 secretion. Obviously, further investigation is necessary for clarifying the optimal method for assessment of change in GLP-1 secretory capacity and its relationship with diseases including coronary artery diseases.

As “residual risks” underlying cardiovascular events under the condition of control of major risk factors, residual dyslipidemia such as small dense LDL-C and oxidized LDL-cholesterol, proatherogenic inflammatory cytokines such as interleukin-1β, and prothrombotic risk have been characterized^6–8^. While GLP-1 plays major roles in the regulation of glucose and lipid metabolism^32,33^, GLP-1 secretion does not appear to correlate with most of the classical coronary risk factors (Table 2). Change in GLP-1 secretion caused by diabetes mellitus is controversial but results of a meta-analysis argue against a significant effect of diabetes mellitus on GLP-1 secretion^34^. In our previous study^17^, AUC-GLP-1 was not correlated with HDL-cholesterol, LDL-cholesterol, fasting plasma glucose, or HbA1c in apparently healthy subjects, while AUC-GLP-1 was weakly correlated with age and systolic blood pressure. In the present study, we found the association of AUC-GLP-1 with increased Gensini score after adjustment of age and hypertension (Table 5). Secretion of GLP-1 from duodenal and ileo-colic enteroendocrine cells is regulated at levels of multiple receptors, delivery of their ligands in the intestinal lumen and intracellular signals downstream of the receptors^10,32,33^. Thus, theoretically, impairment of GLP-1 secretion can occur by multiple mechanisms including non-intestinal causes. In fact, response of plasma GLP-1 to oral glucose loading was found to be reduced in patients with non-alcoholic fatty liver^11^, which is a risk factor of atherosclerotic vascular disease^35^. In addition, Le et al.^36^ recently reported an association of low level of fasting GLP-1 with increased femoral artery intima-media thickness in patients with new-onset diabetes. The findings indicate the possibility that impaired GLP-1 secretion is one of “residual risks” of atherosclerotic vascular disease.

There are limitations in the present study. First, a causal relationship between low response of GLP-1 to oral glucose loading and increased Gensini score could not be proved by the cross-sectional analyses in the present study. A longitudinal study is necessary for clarifying this issue. Second, it is not clear whether glucose is better than other compounds as a trigger of GLP-1 secretion to determine the capacity of GLP-1 secretion. Not only glucose influx via glucose transporters but also activation of fatty acid receptors and bile acid receptors, binding of amino acids to calcium-sensing receptors, and amino acid influx via a sodium-amino acid co-transporter are involved in stimulation of GLP-1 secretion^10,37,38^. Thus, it is unlikely that oral glucose loading is the best stimulus for total capacity of GLP-1 secretion. Third, the number of study subjects was relatively small, and the statistical power was limited. Finally, all of the subjects in the present study were Japanese. In light of the reported ethnic difference in GLP-1 secretion^39^ and the reported effects of a GLP-1 analogue on cardiovascular events^40^, the present findings may not be extrapolated to all ethnic groups.

In conclusion, the results of the cross-sectional analysis in the present study indicate that low response of GLP-1 secretion to oral glucose administration is independently associated with atherosclerotic coronary stenosis. A causal relationship between reduction in GLP-1 secretory capacity and increased coronary stenosis remains to be confirmed by longitudinal analysis.

## Supplementary Information

Supplementary Information.
