# Supplementary material for: Reduction in GLP-1 secretory capacity may be a novel independent risk factor of coronary artery stenosis
Source: Sci Rep. 2021 Aug 2;11:15578. doi: 10.1038/s41598-021-95065-9 (PMC8329155; doi:10.1038/s41598-021-95065-9)
Supplement: Supplementary file 1 — Supplementary Information. [file 41598_2021_95065_MOESM1_ESM.pdf]

Supplementary Tables

Reduction in GLP-1 secretory capacity may be a novel independent risk factor of coronary artery stenosis.

Chihiro Nagase [1*], Masaya Tanno [1*], Hidemichi Kouzu [1], Takayuki Miki [1], Junichi Nishida [1], Naoto Murakami [1], Nobuaki Kokubu [1], Nobutaka Nagano [1], Ryo Nishikawa [1], Nobuhiro Yoshioka [2], Tohru Hasegawa [3], Hiroyuki Kita [4], Akihito Tsuchida [3], Hirofumi Ohnishi [5], Tetsuji Miura [1,6, ORCID 0000-0001-5024-7085]

[1]Department of Cardiovascular, Renal and Metabolic Medicine, Sapporo Medical University, Japan
[2]Sapporo Circulation Hospital, Japan
[3]Department of Cardiology, JR Sapporo Hospital, Japan
[4]Department of Cardiology, JCHO Hokushin Hospital, Japan
[5]Department of Public Health, Sapporo Medical University, Japan
[6]Department of Clinical Pharmacology, Faculty of Pharmaceutical Sciences, Hokkaido University of Science, Japan

Supplementary Table 1. Multivariate logistic regression analysis for GS ≥ 10

	Crude Model		Model 1		Model 2	
Tertiles of AUC-GLP-1	OR (95% CI)	p	OR (95% CI)	p	OR (95% CI)	p
T1	1.88 (0.88 - 4.01)	0.106	1.67 (0.76 - 3.71)	0.204	1.62 (0.73 - 3.63)	0.238
T2	1.14 (-0.51 - 2.55)	0.757	1.17 (0.51 - 2.70)	0.714	1.23 (0.53 - 2.89)	0.630
T3	1.00 (reference)		1.00 (reference)		1.00 (reference)	

AUC-GLP-1: 120~371 in T1, 380~670 in T2, 674~3,386 in T3

OR = odds ratio, CI = confidence interval. Model 1 was adjusted for sex and age. Model 2 was adjusted for sex, age and the presence of hypertension

Supplementary Table 2. Multivariate logistic regression analysis for GS ≥ 10 : Results by use of fasting GLP-1 level ≤ 1 pmol/l, instead of AUC-GLP-1 < median, as a possible explanatory variable.

Model 1							
	B	SE	Wald χ^2	OR	95%CI		p
Sex	0.44	0.20	4.81	2.43	1.10	5.39	0.028
Age	-0.05	0.02	4.80	1.05	1.01	1.09	0.029
Hypertension	0.34	0.18	3.42	1.98	0.96	4.09	0.064
LDL-C	-0.01	0.01	2.32	1.01	1.00	1.02	0.128
Model 2							
	B	SE	Wald χ^2	OR	95%CI		p
Sex	0.45	0.20	4.84	2.45	1.10	5.43	0.028
Age	-0.06	0.02	6.05	1.06	1.01	1.11	0.014
Hypertension	0.39	0.19	4.24	2.20	1.04	4.64	0.040
LDL-C	-0.01	0.01	2.32	1.01	1.00	1.02	0.128
eGFR	-0.01	0.01	1.77	0.99	0.96	1.01	0.183

OR = odds ratio, CI = confidence interval, LDL-C = LDL cholesterol, eGFR = estimated glomerular filtration rate.

Multivariate logistic analysis for Gensini score ≥ 10 was performed by using sex, age, BMI, HOMA-IR, history of hypertension, LDL-cholesterol, HDL-cholesterol, eGFR, and fasting GLP-1 level ≤ 1 pmol/l as possible explanatory variables, and better fit models were selected by using Akaike Information Criterion. Two better models do not include fasting GLP-1 level as a significant explanatory variable.

Supplementary Table 3. Baseline characteristics of patients grouped according to fasting GLP-1 level being > 1.0 or ≤ 1.0 pmol/l.

	Fasting GLP1 > 1.0 (n=48)	Fasting GLP1 ≤ 1.0 (n=125)	p
Age (years)	65.3 ± 10.1	67.0 ± 8.2	0.249
Male (%)	31 (64.6)	91 (73.8)	0.289
BMI (kg/m^2)	24.2 ± 3.6	24.0 ± 3.6	0.828
sBP (mmHg)	120.0 ± 19.5	124.6 ± 16.2	0.111
Hypertension (%)	27 (56.3)	87 (69.6)	0.097
fasting PG (mg/dl)	91.5 ± 8.9	89.9 ± 10.9	0.364
AUC-PG (a.u.)	1854 ± 370	1845 ± 394	0.894
Fasting IRI (μIU/ml)	5.9 ± 3.5	5.2 ± 3.6	0.290
AUC-IRI (a.u.)	694 ± 555	542 ± 404	0.049
Fasting GLP-1 (pmol/l)	2.5 (2.3 - 3.2)	1.0 (1.0 - 1.0)	<0.001
AUC-GLP-1 (a.u.)	679 (449 - 1090)	377 (212 - 746)	<0.001
LDL-C (mg/dl)	106 ± 30	111 ± 28	0.333
HDL-C (mg/dl)	48 ± 11	51 ± 13	0.117
Triglyceride (mg/dl)	124 ± 55	132 ± 64	0.419
S-Cre (mg/dl)	0.84 ± 0.23	0.85 ± 0.21	0.753
eGFR (mL/min/1.73 m^2)	68.6 ± 18.4	67.4 ± 16.6	0.664
HbA1c (%)	5.7 ± 0.4	5.8 ± 0.4	0.408
U-Alb/U-Cre (mg/gCre)	8.3 (4.0 - 22.3)	12.8 (4.4 - 180.0)	0.090
Matsuda-DeFronzo index	8.5 ± 7.0	10.6 ± 10.0	0.182
HOMA-IR	1.4 ± 0.9	1.2 ± 0.8	0.196
HOMA-β	76.3 ± 43.0	77.7 ± 54.2	0.869
Gensini Score	7.5 (1.8 - 24.0)	11.5 (5.0 - 26.0)	0.241

sBP = systolic blood pressure, BMI = body mass index, PG = plasma glucose, IRI = immunoreactive insulin, LDL-C = LDL cholesterol, HDL-C = HDL cholesterol, S-Cre = serum creatinine, eGFR = estimated glomerular filtration rate, HbA1c = glycol-hemoglobin A1c, U-Alb/U-Cre = ratio of urinary albumin to urinary creatinine. Data are means ± SD or medians (interquartile ranges).
